# Hydrolyzation of snail *(Achatina fulica)* meat with rice water as novel probiotic supplements for animal feed

**DOI:** 10.14202/vetworld.2022.937-942

**Published:** 2022-04-15

**Authors:** Ujang Suryadi, Rosa Tri Hertamawati, and Shokhirul Imam

**Affiliations:** Department of Animal Science, Politeknik Negeri Jember, Mastrip Street PO. BOX 164, Jember, East Java, Indonesia

**Keywords:** bile, pH, probiotics, snails, supplements

## Abstract

**Background and Aim:**

Snail meat and digestive tract hydrolyzate fermented with a consortium of preserved rice water microorganisms could serve as new sources of probiotics. Microorganisms from the examined feed supplement were isolated, identified, and characterized for resistance at low pH and with bile salts. The study aimed to determine the potential hydrolysate of the snail meat and digestive tract as a novel probiotic supplement for animal feed at various pH values and Oxgall.

**Materials and Methods:**

The submerged fermentation method was conducted for 21 days to examine the novel probiotic that originated from snail microorganisms in the hydrolyzed liquid fermented by finely ground snail meat and the digestive tract. The microorganisms in the hydrolyzate were isolated by a spread plate method, while the potential of the probiotic hydrolyzate was tested for resistance to pH values of 2, 2.5, 3, and 4, as well as resistance to bile salts at Oxgall concentrations of 0.2%, 0.3%, 0.5%, 1%, and 2%.

**Results:**

The hydrolyzate profile of snail meat and digestive tract contained five isolates of lactic acid bacteria that could serve as potential probiotics.

**Conclusion:**

The application of fermentation technology using a consortium of preserved rice water microorganisms can convert snail meat and the digestive tract into novel probiotic products that could be utilized in feed supplements.

## Introduction

The processing of snail meat (*Achatina fulica*) for chicken feed has been widely carried out using various methods such as cooking [1] and conversion into flour [2]. Although the use of snail meat in laying hens to replace fish meal does not interfere with the production performance, it reduces costs and the adverse impact of agricultural pests. Moreover, its collection and processing can also be a source of income in rural areas [1].

In poultry diets, snails are used as a source of protein because the raw meat contains 81.96% water, 11.3 g/100 g crude protein, 2.56 g/100 g ash, 0.18 g/100 g fiber, and 6.78 g/100 g fat on a dry basis. It also has a high mineral content of amino acids aspartic acid, serine, glutamic acid, glycine, histidine, arginine, threonine, alanine, proline, tyrosine, Valine, lysine, isoleucine, leucine, phenylalanine, cysteine, methionine, and tryptophan [3].

Snails also contain microbiota in their intestines, including a number of lactic acid bacteria (LAB) ranging from 1.03 to 1.30×10^8^ cfu/g, fungi at 7.3×10^7^-1.00×10^8^ cfu/g, total bacteria from 1.00 to 1.50×10^8^ cfu/g, coliform count from 1.68 to 2.20×10^7^ cfu/g, and *Salmonella/Shigella* from 5.2 to 8.2×10^7^ cfu/g. Meanwhile, the microorganisms that could be isolated include *Bacillus subtilis, Staphylococcus aureus, Lactobacillus* spp., *Escherichia coli, Micrococcus luteus*, and *Bacillus cereus*. The fungal isolates included *Aspergillus terrus, Aspergillus fumigatus, Absidia* spp., *Fusarium oxysporum, Eurotium* spp., and *Aspergillus flavus* [4], while the probiotic bacteria include *Aspergillus* and *Bacillus* [5].

The presence of LAB in snails containing probiotic bacteria such as *B. subtilis* and *Lactobacillus* spp. is a multistrain that can be used to produce new probiotics. Moreover, the bacteria that compose probiotics can be multispecies (multistrain) or single-species (single strain) [6]. The presence of *Bacillus* in multistrain probiotics can be used as an alternative for broiler production without an antibiotic growth promoter to improve intestinal health [7].

Furthermore, LAB, which acts as probiotic bacteria, also plays an important role in fermentation. Therefore, the process of using snail meat and the digestive tract through fermentation is carried out using an exogenous starter culture in the form of LAB, which is present in rice water. According to Susilawati [8], six LAB isolates were obtained with Gram-positive and catalase-negative characteristics using rice water fermentation. Microorganisms for fermentation can use microbiota from animals’ intestines because of their coexistence [9].

Fermented products contain functional microorganisms, which include probiotic properties [10], antimicrobial characteristics [11], antioxidant activities [12], peptide production [13], fibrinolytic activity [14], polyglutamic acid [15], and degradation of antinutritive compounds [16]. Apart from being nonpathogenic to animals, the microorganisms used as probiotics are selected based on their viability in the gastrointestinal environment and their ability to withstand low pH values and high bile acid concentrations [6].

In general, products that contain probiotic bacteria are included in dietary supplements [17]. These microbial cells or extracellular microbial products such as food-grade lactic acid, enzymes, amino acids, vitamins, and other pharmaceutical compounds are produced by fermentation techniques [6].

Probiotics are popularly used as bio-ingredients in many functions of fermented foods [18]. According to Lenkey and Soeharsono [19], probiotic microbes are Gram-positive bacteria such as *Lactobacillus* and *Bifidobacterium*. The bacteria used include *Lactobacillus acidophilus, Lactobacillus lactis, Lactobacillus plantarum, Bifidobacterium bifidum, Bifidobacterium thermophilum*, and *Streptococcus lactis*. Other probiotic microorganisms come from *Bacillus, Pediococcus*, and some yeasts [20]. *Lactobacillus* and *Bifidobacterium* anaerobically produce lactic acid, which lowers the pH of the digestive tract and inhibits the development and growth of pathogenic bacteria [10].

Since probiotic strains need to live and colonize the intestinal lumen, it is necessary to examine their resistance to low pH values and bile salts [21]. Therefore, the selection of intestinal microbiota as the origin of probiotics is necessary to obtain strains that live and reproduce in the intestine [21]. Based on the potential of snails, namely, as a source of protein, and their intestines containing probiotic microbiota, the process of fermenting snail meats and their digestive tracts using rice water can produce hydrolysates that are useful as a new source of probiotics.

This study aimed to explore the potential of hydrolyzed snail meat and its digestive tract as a new probiotic for animal feed supplements.

## Materials and Methods

### Ethical approval

All procedures carried out in the study did not involve farm animals that were protected by animal welfare; the object under study only used 2 Kg of snails and did not cause extinction as a natural resource.

### Study period and location

The study was conducted from September to October 2021 at the Microbiology Laboratory of the University of Jember.

### Procedures

This study used snails taken around banana plantations at the Jember State Polytechnic. Snail meats and their digestive tracts were finely ground and then fermented for 21 days with a starter culture of microorganisms from rice washing water which was fermented for 3 days [22] at a pH of 4-5 and a temperature of 33-45°C. According to Wang *et al*. [23], the tolerant fermentation temperature and pH for LAB are 30°C and pH 5. Furthermore, palm sugar was also added as a source of carbohydrates which can be used in bacterial nutrition. The hydrolyzation process was carried out by boiling the snails at a temperature of 60-65°C for 3 min. Then the snail meat and its digestive tract were removed and fermented in rice washing water with liquid brown sugar added in a ratio of 1:1:1 for 21 days. Then the hydrolyzate was filtered and LAB was observed.

### Probiotic characteristics test procedure

The LAB isolate as a probiotic candidate from 1 mL of snail and its digestive tract hydrolyzate was collected and grown in 5 mL of *de mann rogosa sharpe broth* media/liquid (MRS, Merck, Germany) aseptically. The bacterial cultures were incubated (Raypa, Spain) at 30°C for 48 h. Subsequently, 1 mL was taken and re-grown into 5 mL of liquid MRS media and was incubated at 30°C for 48 h. This last culture was tested for its probiotic resistance to gastric acid and bile salts.

### LAB isolate resistance to gastric acid

The survival test under high acidity conditions was carried out by taking 0.1 mL of the probiotic bacterial suspension inoculated into 2 mL of liquid MRS (Merck, Germany) at pH 2.5, which was adjusted by adding 0.1 N HCl (Merck, Germany). Then, it was incubated (Raypa) at 37°C and the total number of bacteria was calculated using the total plate count (TPC) method at 0, 4, 8, and 24 h of incubation.

### Resistance to bile salts

The survival rates of the strains were estimated at different bile salt (Sigma-Aldrich) concentrations of 0.3, 0.5, 1, and 2%, respectively. Subsequently, the bacterial cells were harvested as described above and treated with different bile salt concentrations. After incubating for 4 h at 37°C, the results were assessed in terms of viable cell counts and compared with controls.

### Statistical analysis

The parameters observed were the type of isolate and the identification of microbes in the snail and digestive tract hydrolyzate and isolates from gastric acid and bile salts.

## Results and Discussion

**Figure-1:**
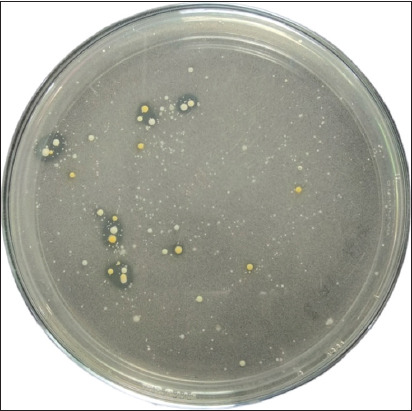
Lactic acid bacteria colony with clear around the colony.

The LAB content was determined based on the formation of a clear zone around the bacterial colonies (Figure-1) and the number of bacteria was calculated using the TPC method. The analysis of LAB colonies from preserved rice washing water revealed three different colonies (Table-1), while the TPC obtained 5×10^7^ CFU/ml.

**Table 1:** Characteristics of lactic acid bacteria colonies from rice washing water.

Colony	Characteristics				

	Colony form	Colony edge	Colony surface	Top color	Bottom color
A1	Circular	Entire	Convex	Milky white	Creamy white
A2	Circular	Entire	Convex	Yellow	Yellow
A3	Circular	Entire	Convex	Creamy white	Cream

Using the fermentation method, an artificial starter was added for regulation. Meanwhile, the microorganisms involved in the natural fermentation process mainly came from raw materials and the surrounding environment [24]. The development of starter cultures for the fermentation of the snail and the digestive tract was adjusted to the desired characteristics to ensure quality and ease in the process. Similarly, Sun *et al*. [25] have used LAB as a starter culture to produce various fermented meat products.

The process of meat fermentation involves a complex ecosystem of microorganisms, which change its composition and characteristics. Various microorganisms such as bacteria (especially LAB), yeast, and fungi [26] are involved in this process. Based on the analysis of LAB colonies in the snail and digestive tract fermented with rice washing water, five colonies with different characteristics (Table-2) and a total of 3×10^4^ CFU/mL were obtained.

**Table 2:** Colonial characteristics of fermented meat and snail digestive tract in fermented rice washing water.

Colony	Characteristics				

	Colony form	Colony edge	Colony surface	Top color	Bottom color
AB1	Circular	Entire	Convex	Milky white	Cream
AB2	Circular	Entire	Flat	Clear white	Cream
AB3	Circular	Entire	Raised with concave beveled edge	Milky white	Cream
AB4	Circular	Entire	Convex	Cream	Yellow cream
AB5	Circular	Entire	Convex	Creamy white	Cream

### LAB probiotic potential test

LAB become probiotics when they have specific characteristics and the viability of the final product remains high in line with the provisions of a probiotic (10^7-9^ CFU/g product) [21]. The amount of LAB, which is 3×10^4^ CFU/mL, in the snail, and digestive tract is lower than the minimum requirement. However, it can be increased by the presence of nutritional supplements derived from synbiotics, which is a combination of probiotics and prebiotics. Prebiotics are selective feed ingredients needed for the growth and activity of bacteria present in the hydrolyzate of snails and their digestive tracts which are fermented with rice washing water. According to FAO [6], they are selective fermentative agents that allow specific changes in the composition and activity of the gastrointestinal microbiota that confers benefits to the host.

Probiotics without a source of nutrition in the form of prebiotics cannot colonize efficiently in the digestive system. Beneficial prebiotics, especially oligosaccharide and disaccharide products, are important food sources that allow probiotics to last longer in the digestive system. The previous study conducted by Patterson *et al*. [27] stated that the use of prebiotics containing xylose, fructose, galactose, mannose, and glucose might be promising.

**Figure-2:**
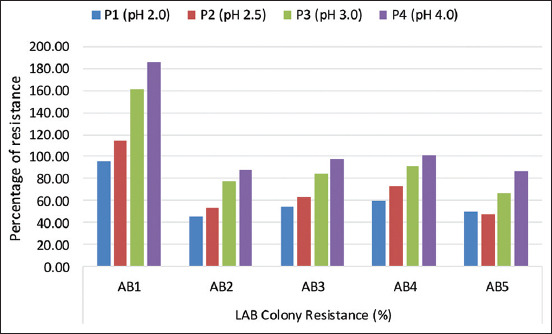
Percentage of five lactic acid bacteria isolates resistance at several acidic pH.

Furthermore, LAB is probiotics when they grow at low pH and bile salt levels. In this study, the five LAB isolates obtained were examined to determine the probiotic potential of each isolate at several pH values, 2, 2.5, 3, and 4 (Figure-2). The results showed that AB1 isolates had the highest percentage of resistance at all test pH values, 95.4% (pH-2), 114.3% (pH-2.5), 161.7% (pH-3), and 186.1% (pH-4). However, the highest percentage of resistance among almost all isolates was observed at pH 4.

**Figure-3:**
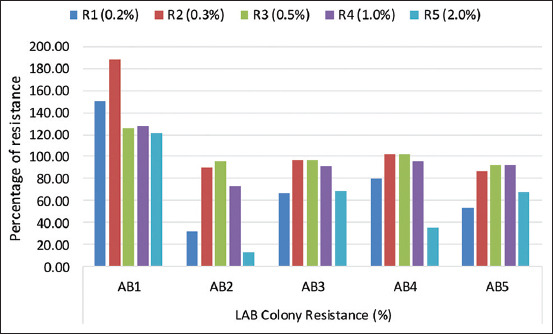
Percentage of five lactic acid bacteria isolates resistance at several concentrations of bile salts.

The resistance test of five LAB isolates was carried out at several concentrations of Oxgall, 0.2%, 0.3%, 0.5%, 1%, and 2% (Figure-3). The results showed that isolate AB1 had a high percentage of resistance at all test concentrations, 149.8% (0.2%), 187.5% (0.3%), 125.9% (0.5%), 127.5% (1%), and 120.8% (2%).

A more than 100% percentage value indicates that the probiotic bacteria can survive and grow in an atmosphere of low pH and high bile salt conditions. Hill *et al*. [10] stated that as a probiotic isolate, for LAB to survive and grow in the digestive tract, they need to pass through various stressful environmental conditions. These include the movement of bacteria from the upper part of the intestinal tract where bile is secreted to the intestine. The ability to survive from bile exposure is one of the criteria commonly used to select potential probiotic strains. This is because bile is a major challenge for bacteria entering the gastrointestinal tract [6]. Therefore, the LAB isolates are assumed to be flexible species that can adapt and synergize with the environment.

Liquid isolates from fermented snail and the digestive tract showed good tolerance at low pH (Figure-2). There were different levels of resistance to bile salts from the five isolates (Figure-3) due to the expression of bile resistance-associated proteins in LAB cells [28]. Furthermore, the isolates also varied in their survivability under acidic conditions due to a species and/or strain-dependent acid tolerance mechanism [29] with certain bacterial proteins that can confer resistance [30]. This is in line with the study of Guo *et al*. [31], who stated that the difference in resistance was caused by variations in certain species and strains and the environmental conditions of bacteria [32]. However, Ramasamy *et al*. [33] stated that there was no relationship between bacterial resistance to acid and its source.

Berrada *et al*. [34] affirmed that approximately 90 min is required for bacteria to enter and leave the stomach. Therefore, isolates selected as probiotics need to survive under gastric acid conditions for at least 90 min. Acid-resistant bacteria have greater resistance to membrane damage due to decreased extracellular pH than non-acid-resistant bacteria. LAB tolerance to acid is also usually used due to its ability to maintain a more alkaline cytoplasmic pH than the extracellular pH [35]. According to Chou and Weimer [36], protease enzymes can affect the growth of LAB at a low pH.

LAB isolates from the snail meat and digestive tract that passed the acid media resistance test were further subjected to the bile salt. The conditions that affect the viability of bacterial cells include a high level of acidity in the stomach and the fluid with a high concentration of salt in the small intestinal tract. Furthermore, high acidity can damage cell membranes and intracellular components, leading to the death of not acid-fast bacteria [37]. The small and large intestines have high concentrations of bile salts, which penetrate the cytoplasmic membrane and damage the cell membranes [38]. Meanwhile, probiotic microbes in the stomach need to be tolerant of pH 3 [39] or a minimum of pH 4, as the pH of the gastric mucus layer [40] and at a minimum bile salt concentration of 0.3% [41].

In this study, it was discovered that five LAB isolates grew at different bile salt concentrations. Gilliland *et al*. [42] stated that a 0.3% concentration of bile salts is a critical and high enough value for selecting resistant isolates. Subsequently, bile salts are amphipathic compounds, in which one side is soluble in water (polar/hydrophilic) and the other side is insoluble (nonpolar/hydrophobic) [43]. The amphipathic structure causes them to emulsify fats and affect the microorganism life in the digestive tract, especially in the small intestine. The presence of microorganisms in the small intestine can also be called biological detergents, namely, liquids that dissolve phospholipids, cholesterol, and proteins. Since most of these compounds form the cell membrane, they cause the microorganism cell to be destroyed (lysis). High concentrations of bile salts are toxic and very strong antimicrobial substances [44]. Bezkorovainy [45] stated that bile in the small intestine inhibits microbial growth; therefore, LAB, especially *Streptococcus*, as a probiotic, needs to resist bile salts to live and function in the intestine. The ability to survive in high bile salt concentrations is a prerequisite for bacterial isolates to form colonies and carry out metabolic activities in their hosts.

## Conclusion

The study on the potential of novel probiotics from the digestive tract of fermented meat and snails using a rice water washing consortium containing five LAB isolates that have the potential to become new probiotics that are resistant at pH values of 2.0, 2.5, 3.0, and 4.0 and bile salt concentrations of 0.2%, 0.3%, 0.5%, 1%, and 2%. However, the number of probiotic bacteria did not reach 10^7-9^ CFU/g, but only 10^3^ CFU/g. Therefore, further study is needed to increase the number of prebiotic bacteria by providing prebiotics as synbiotics.

## Authors’ Contributions

US: Designed the study. RTH and SI: Made the probiotic composition. US and SI: Examined the samples in the laboratory. All authors drafted and revised the manuscript. All authors read and approved the final manuscript.
